# Tunable particles alter macrophage uptake based on combinatorial effects of physical properties

**DOI:** 10.1002/btm2.10047

**Published:** 2017-01-19

**Authors:** Anusha Garapaty, Julie A. Champion

**Affiliations:** ^1^ School of Chemical & Biomolecular Engineering Georgia Institute of Technology Atlanta GA 30332

**Keywords:** elasticity, layer‐by‐layer, phagocytosis, polyelectrolytes, shape, size

## Abstract

The ability to tune phagocytosis of particle‐based therapeutics by macrophages can enhance their delivery to macrophages or reduce their phagocytic susceptibility for delivery to non‐phagocytic cells. Since phagocytosis is affected by the physical and chemical properties of particles, it is crucial to identify any interplay between physical properties of particles in altering phagocytic interactions. The combinatorial effect of physical properties size, shape and stiffness was investigated on Fc receptor mediated macrophage interactions by fabrication of layer‐by‐layer tunable particles of constant surface chemistry. Our results highlight how changing particle stiffness affects phagocytic interaction intricately when combined with varying size or shape. Increase in size plays a dominant role over reduction in stiffness in reducing internalization by macrophages for spherical particles. Internalization of rod‐shaped, but not spherical particles, was highly dependent on stiffness. These particles demonstrate the interplay between size, shape and stiffness in interactions of Fc‐functionalized particles with macrophages during phagocytosis.

**Abbreviations**
BSA: bovine albumin serum
CaT: chloramine trihydrate
DTNB: dithio‐bis‐(2‐nitrobenzoic acid)
DTT: dithiothreitol
EDC: N‐(3‐dimethylaminopropyl)‐N‐ethylcarbodiimide
FITC: fluorescein isothiocyanate
IgG: immunoglobulin‐G;
IPA: isopropyl alcohol
NHS: N‐hydroxysuccinimide
PAA: polyacrylic acid
PDH: pyridine dithioethylamine hydrochloride
PEI: polyethylenimine
PS: polystyrene
PVA: polyvinyl alcohol
PVP: poly(vinylpyrrolidone)
THF: tetrahydrofuran.

## 1 Introduction

Polymeric micro and nanoparticles are useful carriers for drugs and vaccines.^1^, ^2^ In the body, particles come into contact with macrophages of the reticuloendothelial system leading to their internalization.^3^ In many applications, uptake of particles by macrophages affects their ability to deliver therapeutics to the target cells, such as in the case of cancer.^4^ However, in other uses, intracellular delivery of therapeutics is dependent on macrophage uptake in vaccines or diseases involving macrophages.^5^ In some diseases, such as rheumatoid arthritis, macrophages are the target cell for therapeutic particle uptake since they are the local and systemic amplifiers of inflammation.^6^ As such, research efforts have focused on identifying crucial physico‐chemical properties that are capable of enhancing drug delivery. Properties such as size,^7^, ^8^, ^9^ surface chemistry,^9^, ^10^, ^11^, ^12^, ^13^ shape,^14^, ^15^, ^16^ and elasticity^4^, ^17^, ^18^, ^19^, ^20^, ^21^, ^22^ have been shown to affect particle fate and function.

The choice of particle size is dependent on the in vivo application^23^ (e.g., cancer) or the type of the cell being investigated in vitro.^7^ In macrophage cell lines, microparticles in the 2–3 µm range have been shown to exhibit enhanced attachment and phagocytosis based on number of contact points of particle with cell.^8^, ^9^ Carboxylated, 500 nm polystyrene (PS) particles were found to alleviate in vivo conditions like myocardial infarction and experimental autoimmune encephalomyelitis when taken up by inflammatory monocytes.^24^ The surface chemistry of a particle is also crucial, as it influences the type and structure of adsorbed serum proteins on the surface, thus influencing particle uptake.^10^, ^11^ Polyethylene glycol (PEG) or other stealth coatings can reduce interactions with cells.^12^, ^25^, ^26^ In contrast, modification of particle surfaces with ligands for specific cell surface receptors, such as the Fc receptor for macrophages, promotes uptake of particles.^27^ Significant attention has been paid to understanding the role of shape in cellular interactions. High aspect ratio particles (>20) have reduced phagocytic uptake.^14^ Similarly, long and flexible filomicelles evaded the reticuloendothelial system and persisted longer in circulation compared to spheres in vivo.^15^ Bio‐inspired red blood cell discoid‐shaped capsule particles also exhibited lower internalization compared to their spherical counterparts in endothelial, breast cancer, and macrophage cells.^16^ As evident in some of the shape studies, particle elasticity is an emerging physical property that is currently under active investigation. Its role in cellular interactions is still unclear and depends on the materials, cells, and range of elasticity. For the case of immune cells and macrophages, various studies have shown that hard particles are internalized more than soft particles. Soft poly (l‐glutamic acid)‐CpG capsules (800 nm) associated less with plasmacytoid dendritic cells than hard capsules.^19^ Similar results were observed in the case of tannic acid/polyvinylpyrrolidone (TA/PVPON) soft cubical and spherical capsules,^4^ PEG hydrogel spheres,^21^ polyacrylamide spheres,^20^ and poly(lactic‐co‐glycolic acid)‐PEG lipid disks.^22^ However, in another study, hydrogel particles (∼ 170 nm) of intermediate elasticity were internalized more than those at either extreme.^28^ For the case of cancer cells, softest hyaluronic acid capsules (2.4 µm) exhibited highest association, both internalization and attachment to HeLa cells, compared to stiff capsules of similar size.^17^ Similar results were observed in the case of dextran sulfate sodium salt and poly‐L‐arginine hydrochloride or poly(sodium 4‐styrenesulfonate) and poly(allylamine hydrochloride) based capsules (sizes 4.1 and 4.7 µm), where soft capsules were internalized and transported to lysosomes faster than hard capsules in HeLa cells.^18^ Conversely, PEG hydrogel bare or intercellular adhesion molecule 1 functionalized hard spherical particles (200 nm) were internalized more than soft particles of similar size by 4T1 breast cancer cells.^21^ From these cases, it is clear that elasticity plays a complex role in cellular interactions and that it may be highly dependent on the cell type and the other properties of the particles being studied, such as size, chemistry, or shape.

Most of the studies above have elucidated the effect of either one or two physical properties on cellular interactions. However, as particle synthesis techniques become more advanced, there can be interplay between more than two different physico‐chemical properties on cellular interactions. A recent study has identified synergy between physical (shape), biological cue (antibody display), and route (cellular hitchhiking) for delivery to the lung endothelium.^29^ It is thus crucial to be able to interrogate each of these properties independently of the others. We have previously developed a system where particle size and shape can be decoupled through a soft template, ethanol based layer‐by‐layer (LbL) technique.^30^ In this work, we have expanded the system to tune size, shape, elasticity, and ligand immobilization while retaining the same surface chemistry. We systematically investigated the combined contributions of these properties on cellular interactions during phagocytosis, revealing complex interplay between the properties. Particles varying in multiple physical properties exhibit very different trends during interactions with macrophages. These results can be leveraged to enhance drug delivery to macrophage target cells or avoid macrophage uptake for delivery to other cell types.

## 2 Experimental Section

### 2.1 Materials

Polyacrylic acid (PAA, Mw ∼ 100 kDa), polyvinylpyrrolidone (PVP, Mw ∼ 55 kDa), branched polyethylenimine (PEI, Mw ∼ 120 kDa), tetrahydrofuran (THF), and fluorescein isothiocyanate (FITC) were obtained from Alfa Aesar. Dimethyl sulfoxide (DMSO), isopropyl alcohol (IPA) was purchased from British Drug House (BDH) and pyridine dithioethylamine hydrochloride (PDH) was obtained from Chem‐Impex International Inc. (Wood Dale, IL). Dithio‐bis‐(2‐nitrobenzoic acid) (DTNB) and dithiothreitol (DTT) were received from G Biosciences. Chloramine trihydrate (CaT) was received from Acros Organics. N‐hydroxysuccinimide (NHS), N‐(3‐dimethylaminopropyl)‐N‐ethylcarbodiimide (EDC), polyvinylalcohol (PVA, hydrolyzed degree 99+%, Mw = 85–124 kDa) and ethanol were purchased from Sigma‐Aldrich. All materials were used as received. Ultrapure water was obtained from a Millipore Synergy UV system (18.2 MΩ). Trypan blue and Triton X‐100 surfactant were purchased from Corning Cellgro and EMD Millipore, respectively. Bovine albumin serum (BSA) was obtained from Fischer Scientific. PS carboxyl functionalized particles (3 µm, 6 µm diameter) were purchased from Polysciences. The stock solutions of PEI (5 mg/mL), PVP (5 mg/mL) and PAA (5 mg/mL) were prepared in 80% ethanol.

### 2.2 Cells

J774 macrophages (American Type Culture Collection [ATCC]) were grown in Dulbecco's Modified Eagle's Medium (DMEM) (ATCC) at 37°C in a humidified atmosphere containing 5% CO_2_. The media was supplemented with 10% fetal bovine serum (Seradigm) and 1% penicillin/streptomycin (Amresco). The cells were used between passages 4 and 15.

### 2.3 Fabrication of rod shaped particles

Our previously reported method was used to fabricate rod shaped particles.^31^ Briefly, 1 mL of 2.6 wt% spheres was suspended in PVA solution (0.1 g/mL) and the solution was dried to yield a film of thickness of ∼70 µm. The film was stretched in an oil bath maintained at 120°C to an aspect ratio of 2.5. The film was cooled to room temperature and the particles were extracted from the film by heating the film in a 30% IPA‐water solution maintained at 65°C. The particles were then centrifuged at 2,500 g using an Allegra X‐15R Centrifuge (Beckman Coulter) for 15 min. This process of washing was repeated six times and the particles were dispersed in 80% ethanol.

### 2.4 FITC modification with amine

FITC was dissolved in DMSO to 0.2 M. EDC and NHS were added to the mixture at a molar ratio of 1.4:1.2:1 (EDC: NHS: FITC). The reaction was carried out for 10 min and 1,6 diamine hexane was added to the mixture at a molar ratio of 0.9:1 (1,6 diamine hexane: FITC). The reaction was protected from light and carried out overnight. The solution was centrifuged to remove any precipitates.

### 2.5 Functionalization of PAA with FITC and thiol (PAAm)

PAA (36 mg/mL) was dissolved in 50% DMSO. EDC (43 mg) was added to the mixture followed by NHS (26 mg) and allowed to react for 10 min. PDH (44 mg/mL) and FITC modified with amine (0.2 M) were added simultaneously to the above mixture and the reaction was allowed to proceed overnight. The resulting mixture was then dialyzed against 50% ethanol with 0.025 vol/vol% β‐mercaptoethanol overnight. The functionalized polymer was finally reduced with DTT (1 M) for at least 3 hr. For the non‐fluorescent version of PAA with thiol, the same protocol was used without FITC.

### 2.6 LbL assembly on particles and capsule fabrication

PS particles (150 µL of 2.6 wt% suspension) were dispersed in 4 mL of 80% ethanol. To this, 1 mL of each adsorbing polyelectrolyte layer from the stock solution was added. The suspension was sonicated for 10 min and centrifuged at 1,000 g for 10 min. The polyelectrolyte adsorbed particles were then washed in 80% ethanol through sonication and collected through centrifugation. In this way (PVP/PAA)_2_PEI(PAAm/PVP)_2_PAA coated particles were obtained. Particles were cross‐linked using 1 mM CaT solution for 2 min. Polyelectrolyte coated particles were suspended in 750 µL THF for 2 min to remove the PS core and form capsules. The capsules were then centrifuged at 1,000 g for 2 min and this process was repeated once. The capsules or coated particles retaining their core (referred to as core‐shell particles) were suspended in phosphate buffered saline (PBS) for cell studies.

### 2.7 IgG functionalization of particles

Particles (core‐shell or capsule) were coated first with BSA, using 10 times excess of the surface monolayer saturation as calculated from the manufacturer's protocol. The particles were incubated with BSA (1 mg/mL) in PBS for 2 hr, washed three times in PBS and collected by centrifugation at 1,000 g for 2 min. BSA coated particles were incubated for 1 hr with rabbit anti‐BSA IgG antibody (ThermoFisher Scientific) at a mass ratio of 1:1 (BSA: IgG). The IgG functionalized particles were washed three times with PBS. To confirm the presence of IgG, particles were incubated for 30 min with FITC goat‐anti‐rabbit IgG (BD Life Sciences) and washed with PBS three times. The washed particles were assessed by flow cytometry (Accuri C6, Beckton Dickenson Biosciences).

### 2.8 Particle imaging

Fluorescent images of particles were taken using a Zeiss Axio Observer Z1 inverted microscope. The ability of spherical capsules to conform to a circle was measured using the circularity function in ImageJ software. Transmission electron microscopy was carried out using JEOL 100CX‐II microscope operating at 100.0 kV. Particles were dropped on a Cu grid 400 mesh (Electron Microscopy Sciences) and allowed to settle for 10 min. Any excess solution was wiped off and the grid was left to dry overnight before imaging.

### 2.9 Stability of capsules in media

Particles (10^6^) were incubated with DMEM media supplemented with 10% FBS for 48 hr. The particles were gated by their forward and side scatter and retained similar fluorescence. The particles were then counted at 0, 24, and 48 hr by flow cytometry. Experiments were performed in triplicate and data represented as the mean ± SD.

### 2.10 Cytotoxicity assay

Cells (10,000 per well) were plated in triplicate in a 96 well plate. Particles at the ratio of 10:1 particles: cell or 10 µL of sterile water were added to the wells and incubated for 24 hr. Controls for maximum LDH activity were also run according to the manufacturer's instructions (Pierce LDH Cytotoxicity Assay, Thermo Scientific). Each sample condition (50 µL) was transferred to a 96 well plate and 50 µL of reaction mixture was added to each well and mixed. The reaction was protected from light for 30 min and 50 µL of stop solution was added to each well. The absorbance values were measured at 490 and 680 nm using Biotek Synergy H4 Multimode plate reader (Biotek Instruments Inc., Winooski, VT) and reported as percentage cytotoxicity according to the manufacturer's instructions.

### 2.11 Size and zetapotential measurements of capsules

Capsules (10^5^) stored in PBS for 24 hr were dispersed into a counting chamber glass slide and their size distribution was determined using a Cellometer Auto M10 Counter (Nexcelom Biosciences). Zetapotential was determined by measuring the electrophoretic mobility of particles in 10 mM NaCl using a Zetasizer Nano ZS90 (Malvern Instruments Ltd., Westborough, MA). Non‐fluorescent PAAm was used to determine the zetapotential of particles.

### 2.12 Thiol quantification

The thiol content was determined from Ellman's test (Uptima). Briefly, 50 µL of DTNB solution (2 mM DTNB, 50 mM sodium acetate), 100 µL of Tris solution (1 M), and 840 µL water were mixed. To this working solution, either 10 µL of PAAm sample or standard was added. The solution was mixed well and incubated at room temperature for 5 min. The absorbance values were recorded at 412 nm. Based on the calibration curve, the thiol content was computed. The thiol content was estimated for three different batches of PAAm.

### 2.13 Particle association with cells

J774 cells (10^5^ per well) were plated in a 24 well plate overnight. Particles were added to the cells in supplemented media at 10 particles/cell. The cells were incubated with particles for 2 hr. The cells were then washed with cold PBS three times and scraped from wells. Duplicate samples were centrifuged at 3,000 g for 3 min, treated with 200 µL trypan blue, and immediately interrogated on a flow cytometer. Control samples were run at 4°C and corresponding duplicate samples were treated with trypan blue to determine the effectiveness of quenching attached particles. Cell populations were gated and threshold fluorescence was established based on the auto‐fluorescence of J774. Cell events beyond this threshold were reported as particle positive cells. The phagocytic assay was repeated three times using three different batches of prepared particles. Particle quenching by trypan blue was confirmed by incubating 10^5^ fluorescent particles in 200 µL of trypan blue and interrogating immediately on a flow cytometer.

### 2.14 Confocal microscopy

Cells (10,000 per well) were plated in a Lab‐Tek II chamber slide (Thermo Fisher Scientific) and allowed to adhere at least 6 hr. Particles were added to cells at 10 particles/cell and incubated with cells for 1 hr. The cells were washed with cold PBS twice and fixed with 3.7% formaldehyde for 15 min at room temperature. The cells were then washed with PBS once quickly and two times for 5 min. Cells were permeabilized with 1% Triton–X 100 in PBS for 15 min at room temperature and washed three times with PBS. Phalloidin‐rhodamine (Sigma Aldrich) was used to stain the actin at a volume dilution of 1:500 for 15 min at room temperature. The cells were washed three times before imaging using a Zeiss LSM 700‐405 Confocal Microscope (Carl Zeiss Microscopy, LLC, Thornwood, NY).

### 2.15 Statistical analysis

Two‐way ANOVA was performed to analyze the effect of size and stiffness or shape and stiffness on internalization. When the interaction parameter (size × stiffness or shape × stiffness) was significant, post hoc testing was performed. *p* < .05 was considered to be statistically significant in all analysis. Statistics were performed using the GraphPad Prism6 software.

## 3 Results and Discussion

### 3.1 Fabrication of capsules

**Figure 1:**
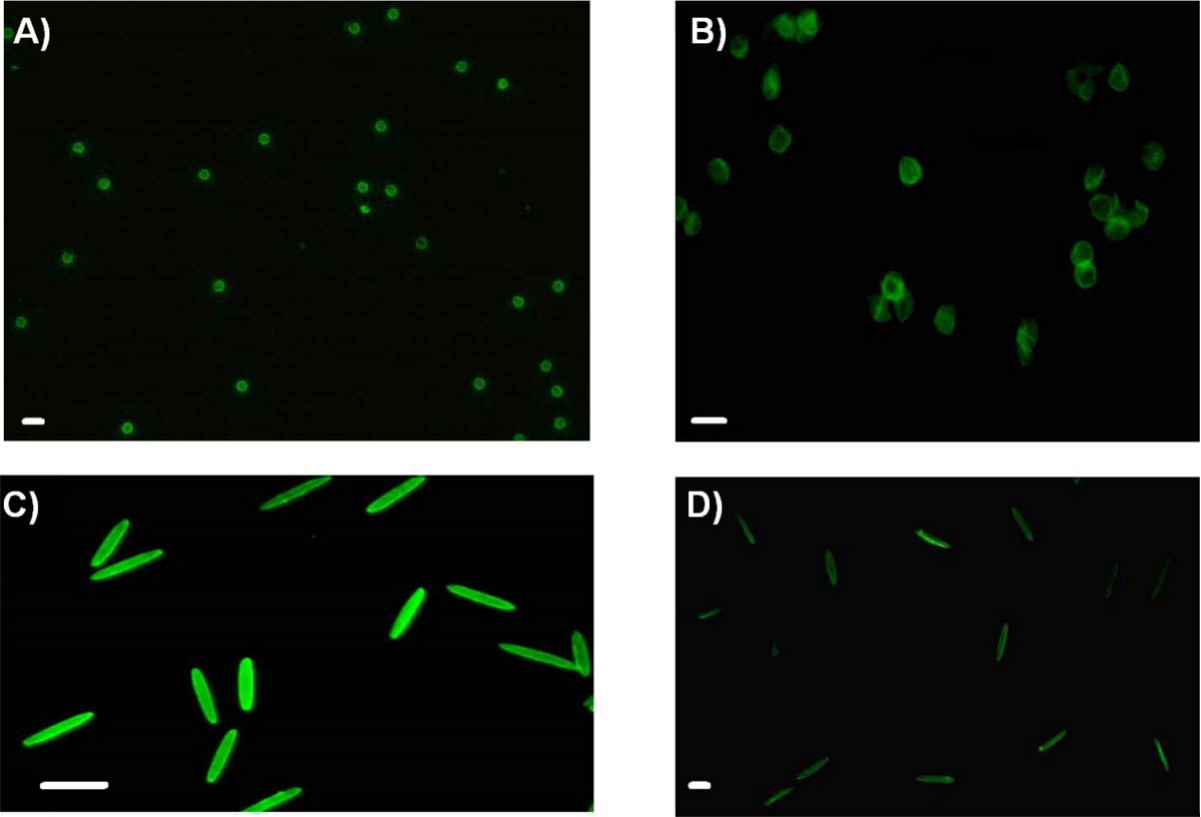
Fluorescence images of particles. (A) 3 µm spherical capsules (scale bar 5 µm), (B) 6 µm spherical capsules (scale bar 10 µm), (C) rod shaped core‐shell particles (scale bar 10 µm), (D) rod shaped capsules (scale bar 5 µm)

PAA, PVP, and PEI were chosen due to their solubility in ethanol. The choice of the solvent has been shown to be critical to prevent aggregation of particles during LbL on soft templates.^30^ Despite the low polarity of ethanol, hydrogen bonding between PVP and PAA is strong enough to sustain LbL on PS particles. PEI is used as a middle layer to impart high charge density for subsequent layer deposition. Zeta potential was used to monitor the progress of LbL on the particles. The zeta potential values changed as a function of the charge of the end group in the depositing polyelectrolyte (Figure S1). Depending on the choice of polyelectrolyte, the interactions of the hydrogen‐bonded layers are susceptible to disintegration at physiological pH.^32^, ^33^ At a pH above the pKa of PAA, 4.2,^34^ its ionization disrupts hydrogen bonding, rendering capsules unstable. In such cases, crosslinking of hydrogen bonded layers through amide or thiol chemistry is commonly used to endow stability to capsules for biological applications.^35^ Hence, we functionalized PAA with thiols in the presence of EDC and NHS yielding PAA with 10 mol% of thiol containing units. This polyelectrolyte was further modified with FITC (PAAm) to fluorescently label the capsules. Functionalization of PAA did not affect the build‐up of layers, as seen from the zeta potential (Supporting Information Figure S1). These layers were cross‐linked with CaT which has been shown to oxidize thiols controllably.^33^ The layered templates (PVP/PAA)_2_PEI(PAAm/PVP)_2_PAA were incubated in THF to remove the PS core. The removal of the core has been shown to reduce the elasticity of spherical TA/PVPON capsules when compared to their core‐shell.^4^, ^36^ Hydrogen bonding between the layers of capsules was also shown to substantially reduce elasticity in comparison to traditional LbL capsules based on electrostatic interactions.^36^ Figure 1 shows fluorescence images of the particles. The capsules retain the morphology of the template and 6 µm capsules appear more deformable than 3 µm capsules. The core removal was further confirmed by TEM (Figure S2). The size distribution of the hollow spherical capsules (3 and 6 µm) in PBS was found to be similar to that of corresponding core‐shell particles (Figures S3 and S4). This confirms the conservation of size and the dispersability of the capsules. Rod shaped core‐shell particles and corresponding capsules were also made using the same LbL technique on rod‐shaped templates (12 ± 0.2 µm in length and 2.5 ± 0.23 µm in width) stretched from 3 µm PS spheres, thereby conserving particle volume. The rod capsules retain the shape of the template as seen in Figure 1C,D. Through this LbL technique, we fabricated core‐shell particles (3 µm sphere, 6 µm sphere, rod) and corresponding capsules (3 µm sphere, 6 µm sphere, rod) of the same surface chemistry yet varying in size, shape, and elasticity. The stability of capsules was tested in biologically relevant media, DMEM supplemented with 10% serum, and they were found to be stable for at least 2 days (Figures S5 and S6). The particles were determined to be non‐cytotoxic to macrophages after incubation for 24 hr (Figure S7).

Isolating the effects of physicochemical properties on cellular interactions is critical to understand the roles that these properties can have when combined. Choice of particle size often depends on the in vivo application.^23^ The advancement of techniques such as particle replication in non‐wetting template, stretching and liquefaction, step flash imprint lithography, and self‐assembly have enabled the fabrication of various non‐spherical particles.^37^ Stiffness of capsules can be altered by varying the number of layers, cross‐linking, choice of material or maintaining/removing the core.^38^ It is crucial to maintain similar size and shape during these processes while tuning stiffness. Our platform based on the choice of template particle size, method of stretching, and hollow preparation enables control over these properties and can help understand any interplay that exists between size, shape, and stiffness.

### 3.2 Interaction of particles with J774 macrophages

When particles are introduced systemically or locally into the body they encounter macrophages.^3^, ^39^ Macrophages are capable of phagocytosing particles through varied mechanisms, though Fc receptor mediated is the most widely studied.^40^ To promote specific particle interactions with macrophage Fc receptors, core‐shell and capsule particles were opsonized with Immunoglobulin‐G (IgG). To achieve proper orientation of the IgG Fc domains to Fc receptors, the particles were first coated with BSA followed by incubation with anti‐BSA IgG. IgG functionalization of particles was confirmed by flow cytometry (Figures S8 and S9).

Recently, the density of Fc‐ligand functionalization on spherical particles of different sizes has been shown to affect overall phagocytosis by macrophages.^41^ The influence of such biological cues was shown to be negligible for spheres of sizes beyond 3 µm. Hence, for our 3 and 6 µm spherical particles, any effects of Fc‐ligand density on phagocytosis would be negligible. For the case of rods, the influence of Fc density on shaped capsules and corresponding core‐shells has been eliminated by equivalent antibody functionalization (Figure S9).

The effect of various particle physico‐chemical properties size, shape, surface chemistry, and stiffness has been extensively studied to tune phagocytic interactions between macrophages and particles.^8^, ^9^, ^14^, ^20^ Attachment and internalization are the first two steps of the phagocytic process. To understand the independent effect of particle properties on internalization at 37°C we used trypan blue quenching of FITC to differentiate between cells with membrane bound particles from cells that had completely internalized particles. Trypan blue quenched the fluorescence of particles by at least an order of magnitude (Figure S10). To confirm the applicability of this technique to differentiate between attached and internalized particles with cells, particles were incubated with macrophages at 4°C, which prevents energy‐dependent internalization processes including phagocytosis. As with free particles in solution, trypan blue also quenched the fluorescence of all the different types of particles attached to macrophages at 4°C, establishing the ability to distinguish between attached and internalized particles at 37°C (Figure S11).

**Figure 2:**
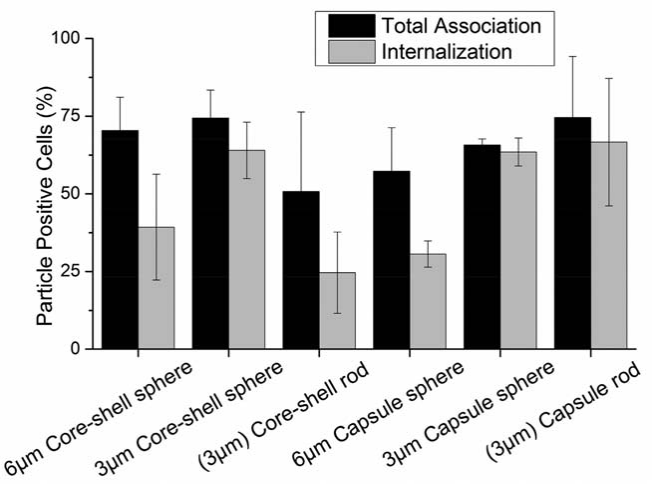
Percentage of J774 macrophages that associated with particles of varied physical properties, as measured by cell fluorescence: total association (black), internalized (gray). Data represented as mean ± SD (*n* = 3)

The effect of IgG functionalized capsule or core‐shell particles on total association (attached and internalized) and internalization by macrophages was evaluated after 2‐hr incubation with cells at 37°C. The dependence of total association and internalization by macrophages due to varied particle physical properties is summarized in Figure 2. There was no statistical difference between the total association of core‐shell particles and capsules of any kind.

Despite similar total association for particles of varying physical properties—size, shape, and stiffness, the internalization trend is different when any of these physical properties is altered. However, from one‐way ANOVA analysis it is unclear if there exists any interplay between physical properties. For example, it is not clear if the effect of size for a spherical particle on phagocytic interaction is the same across varying stiffness. Likewise, the effect of altering shape from sphere to a rod on such interactions across varying stiffness is also unknown. Even when particles being compared appear to have only one physical parameter varied between them, the physical parameters size, shape, and stiffness are inherently related to each other. Therefore, two‐way ANOVA was performed to delineate the interplay between any two physical parameters on internalization by macrophages.^42^

### 3.3 Interplay between size and stiffness of spherical particles

**Figure 3:**
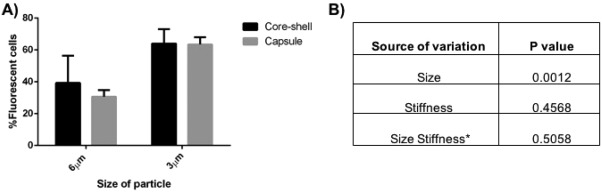
(A) Role of size and stiffness on internalization: core‐shell (black), capsule (gray). Data represented as mean ± SD (*n* = 3). (B) *p* values calculated for each source of variation by two‐way ANOVA

The interdependence between size and stiffness on internalization of spherical particles is shown in Figure 3A. The internalization enhancement seen with decreased size is greater for capsules than for stiffer core‐shell particles. This could be due to differences in the sizes or differences in the stiffness of particles. Two‐way ANOVA analysis, summarized in Figure 3B, determines that size (*p* = .001) plays a dominant role over stiffness (*p* = .4568) for these spherical capsules in affecting internalization. There is no significant interaction between size and stiffness on influencing internalization by macrophages (*p* = .5058). In a recent study, measurement of single‐event engulfment of 3 µm silica spheres by RAW macrophages exhibited ∼ 2.8 times slower engulfment time than 1.85 µm spheres.^43^ This could be attributed to the energetics associated with deformation of the macrophage membrane during internalization of particles. Larger spherical PS particles (3 µm) have been reported to be taken up more slowly compared to 1.5 µm sized particles, suggesting that higher energy requirement for membrane deformation of large particles could abate uptake.^44^ In addition, modeling of flexible nanoparticle interactions with membranes has identified the relative size between the particle and the interacting cell as a parameter in dictating the ability of the cell to execute complete wrapping of the particle.^45^ The energy required for wrapping relatively larger particles is higher, which suggests this is the reason for the dominant effect of size in internalization.

**Figure 4:**
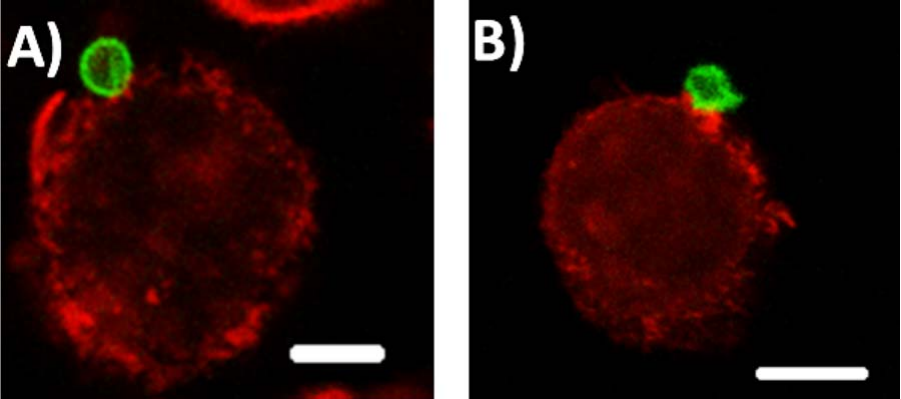
Confocal microscopy images from the center of the stack of J774 cells incubated with particles for 1 hr: (A) 3 µm capsule (scale bar 5 µm), (B) 6 µm capsule (scale bar 10 µm)

It has been established that stiffness is inversely proportional to the radius of a thin hollow spherical shell (Supporting Information Section 1), so larger capsules exhibit reduced stiffness.^46^ Analysis of fluorescent images of capsules in solution (such as those in Figure 1) revealed that 6 µm capsules have 67% conformation to a circular shape, suggesting that 6 µm capsules are less stiff than 3 µm capsules, which have 85% conformation. This is also evidenced from confocal microscopy images in Figure 4, where 6 µm capsules are deformed by the cell membrane while 3 µm capsules maintain their spherical shape. However, the stiffness of capsules is also dependent on the choice of polyelectrolyte and the number of the layers that constitute the capsule so a different selection of either of these could alter the results and highlights the difficulty of selecting fabrication conditions and comparing to the literature. For example, capsule elasticity was greater by 3.5–5 times when poly(allylamine hydrochloride) was the choice of polyelectrolyte over poly(diallyldimethyl ammonium) chloride for capsule formation with poly(styrene sulfonate sodium salt).^47^ An increase in the number of layers has been correlated to increased stiffness of capsules in the case of poly(styrene sulfonate)/poly(allylamine hydrochloride) and hyaluronic acid capsules.^17^, ^18^ In addition, it has been suggested through modeling that the stiffness ratio between the particle and the membrane, is critical in dictating either partial wrapping or complete wrapping by the cell.^48^ It is possible that our particular materials system could be beyond this critical ratio for the cell type we are using and thus not influencing the interaction between capsules and the membrane. However, as discussed below, the difference between core‐shell particle and capsule stiffness does play a role in internalization of rod‐shaped particles, which underscores the complexity of the interactions and difficulty in making broad conclusions.

When size and stiffness are altered for a spherical shape, our results show that a larger size and decrease in stiffness reduces internalization but also that altering the size plays a dominant role compared to stiffness in effecting internalization. This could depend on the crucial role of size in governing the number of attachment points of the particle to the cell and the energetics required for engulfment.^8^, ^44^ Altering the stiffness can affect the ability of actin cup formation, leading to reduced internalization.^20^ Though the influence of stiffness in our case is negligible, the adhesion strength between the particle and the membrane for a particular stiffness could be explored with our particles to achieve full or no wrapping by macrophages. Models for relatively stiff particles have established that altering the adhesion strength alone can help achieve either no wrapping or full wrapping.^48^

### 3.4 Interplay between shape and stiffness of particles

**Figure 5:**
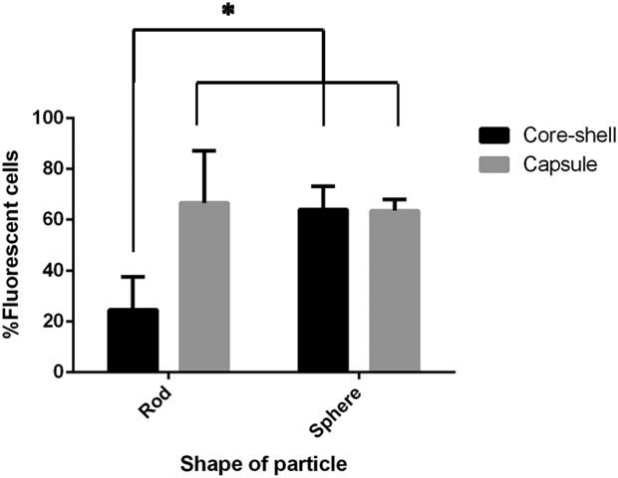
Role of shape and stiffness on internalization: core‐shell (black), capsule (gray). Data represented as mean ± SD (*n* = 3). (*p* = .044 for shape, *p* = .026 for stiffness, *p* = .023 for shape × stiffness, by two‐way ANOVA followed by Sidak's multiple comparison test)

To understand the effect of shape and stiffness, spheres and rods of identical volume were used. As seen in Figure 5, both the shape and stiffness together affect internalization (interaction shape × stiffness *p* = .023). When we look at the individual effects of shape for core shell particles in Figure 5, switching from sphere to rod shape decreases internalization while it has no effect on capsule rods and spheres. Studies have elucidated that shape of a particle can influence attachment to and internalization by a macrophage independently.^49^ Prolate ellipsoids have been shown to achieve maximum attachment compared to sphere and oblate ellipsoids. However, oblate ellipsoids exhibited higher internalization when compared to these two shapes. In another study, antibody‐displaying micro‐rods were shown to exhibit higher attachment and uptake compared to spheres in breast cancer cells.^50^ The data in Figure 2 suggests that the rod shaped core‐shell particles are able to attach to the macrophage but are unable to efficiently convert attachment into internalization. Our prior work with core particles hypothesized this was due to minimized curvature at the point of contact.^14^ Switching the shape from sphere to rod has no effect on internalization of capsules. As evident from the analysis, this difference from core‐shell particles must be due to the combined effects of shape and stiffness. Our results contradict a previous study where PMA spherical capsules were shown to be internalized more than rod shaped capsules.^51^ This could be due to the different size (∼ 300 nm) and mechanism of internalization, or the type of capsules and cells used, which make comparison difficult.

**Figure 6:**
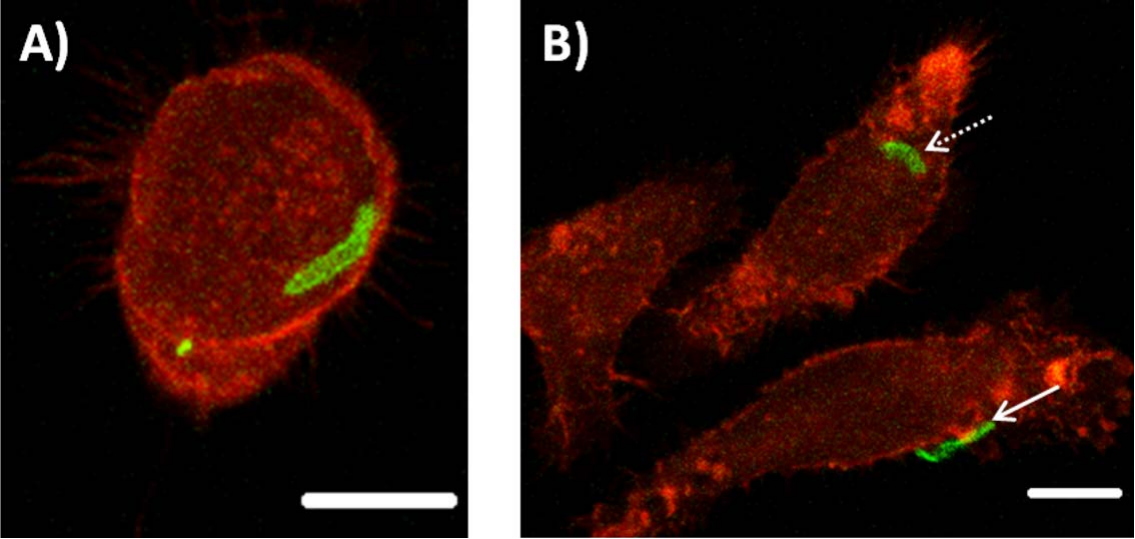
Confocal microscopy images from the center of the stack of rod capsules incubated for 1 hr with J774 cells: (A) bent rod and (B) rod folded in half (dotted arrow), twisted rod (solid arrow), scale bar 10 µm

When we isolate the role of stiffness in these interactions; for 3 µm spheres, altering stiffness seems to have no effect on internalization. However, decreasing stiffness of rods has a significant 2.6‐fold increase in internalization. The reduced stiffness of the rods enables their bending and twisting by the cell on internalization, as seen in Figure 6. The stiffness of anisotropic particles is dependent on their orientation during interrogation methods like atomic force microscopy.^52^, ^53^ In the case of hollow tubes made from glass fiber templates, the stiffness varied with the location of probing on the tube, highlighting the challenges involved in characterizing mechanical properties of anisotropic shapes. Recently, a non‐contact mode of determining axisymmetrical pendant capsule elasticity was developed based on the pendant drop method. This method determines the capsule's resistance to stretching and its bending stiffness from shape and wrinkle analysis during deflation of the capsule.^54^ However, it may not be possible to analyze different geometries through this method. Models to establish the global bending of shaped capsules like tubes is actively being pursued.^55^ Such investigations can help identify the dependence of elasticity on shape.

Varied stiffness has a complex interaction with shape, affecting internalization in the case of rods while having negligible effect on spheres. Particle shape has been widely investigated to alter cellular attachment and internalization of particle based therapeutics.^14^, ^15^, ^49^ Oblate ellipsoids were found to be internalized more than prolate ellipsoids or spheres during phagocytosis.^49^ A 2D model for phagocytosis of non‐circularly symmetric particles predicts similar results for oblate ellipsoids when presented on the flat side to the macrophage membrane.^56^ Despite the usual convention of high particle curvature promoting fastest engulfment, the model suggests that orientations like the flat side of oblate ellipsoids can also promote uptake. The mechanism of this is still to be investigated. In another study, BSA coated poly (allylamine hydrochloride)/poly(styrenesulfonate) bowl‐like microcapsules were found to be internalized greater than spherical microcapsules in smooth muscle and macrophage cells. Bowl‐like capsules were found to mainly attach to cells from the convex‐side, and it was hypothesized that less energy expenditure is required for membrane deformation and that facilitates enhanced uptake of these capsules over spherical shapes.^57^ Though the field does not yet have a unified theory, these studies contribute to the role of shape and orientation of the particle in enhancing uptake.

The effect of particle elasticity on cellular interactions, to a certain extent, shows consensus for immune cells that soft spherical or non‐spherical particles are internalized to a lesser extent than their stiffer controls.^4^, ^19^, ^20^, ^22^ TA/PVPON cubical or spherical microcapsules exhibited less uptake in macrophages compared to their respective stiff counterparts. In addition, no difference in uptake between spherical or cubical stiff core‐shells was reported, highlighting that a cubical shape for stiff core‐shells has no effect on internalization. Reduced stiffness in this case worked in concert with shape (cubical or spherical) to reduce internalization by macrophages, emphasizing the complex interaction that stiffness can have on internalization for different shapes.^4^ In the case presented here, internalization by macrophages is enhanced due to a combination of non‐spherical, rod shape and reduced stiffness. For two particles of the same volume, an increase in surface area increases the probability of adhesion when comparing rods and spheres. This increase in adhesion contact area will also enhance the interaction between the particle and membrane, facilitating full wrapping through adequate compensation of membrane bending.^58^ In addition to shape, reduced stiffness can increase the ability of the macrophage to feel the particle by deformation, causing changes in the local curvature of the particle at the point of attachment.^59^, ^60^ This can facilitate wrapping by the cell membrane, resulting in enhanced internalization of shaped capsules over their corresponding core‐shells. These tunable particles thus highlight the interplay between physical properties size, shape and stiffness to alter cellular interactions.

## 4 Conclusions

We have developed a system where the effect of multiple physical properties on cellular interactions can be evaluated. Using these particles, we have analyzed the effects of size, shape, and stiffness on macrophage interaction and internalization. Though these properties have an individual effect on cellular interactions, the way each of these influence interactions changes when they work in concert with other properties. In our results, it is evident that stiffness behaves in a complex way when combined with size or shape. Increase in size reduces internalization of spherical particles, while changes in stiffness have no effect. However, varying stiffness had a significant effect on internalization for rod shaped particles. These results reveal the complex interplay between physical properties and highlight the challenges in making broad conclusions about the roles of size, shape, and stiffness on phagocytosis, as is evident from the seemingly conflicting reports in the literature. This system can be used to fabricate advanced carriers or understand membrane mechanics and can be leveraged to identify combinations of properties that improve particle‐based delivery systems or reveal new insight into phagocytosis.

## Supporting information

Additional Supporting Information may be found online in the supporting information tab for this article.

**Figure S1** Zetapotential of particles during LbL process. Data represented as mean ± SD (*n* = 3)
**Figure S2** TEM images of capsules. (A) 3µm sphere, (B) rod (scale bars 2 µm)
**Figure S3** Size distribution of 3 µm core‐shell and capsule particles measured in PBS
**Figure S4** Size distribution of 6 µm core‐shell and capsule particles measured in PBS
**Figure S5** Stability of spherical 3 µm capsules in DMEM supplemented with 10% serum. Data represented as mean ± SD (*n* = 3)
**Figure S6** Stability of rod capsules in DMEM supplemented with 10% serum. Data represented as mean ± SD (*n* = 3)
**Figure S7** Cytotoxicity of 3 µm core‐shell and capsule particles to J774 cells following 24 hr incubation. Data represented as mean ± SD (*n* = 3)
**Figure S8** Spherical 3 µm core‐shell and capsule particles after opsonization with anti‐BSA IgG antibody and incubated with secondary antibody to confirm IgG presence: core‐shell (blue), capsule (green), and negative controls incubated with BSA and secondary antibody only, core‐ shell (red) and capsule (brown)
**Figure S9** Rod core‐shell and capsule particles after opsonization with anti‐BSA IgG antibody and incubated with secondary antibody to confirm IgG presence: capsule (brown), core‐shell (blue), negative control incubated with BSA and secondary antibody only: core‐shell (purple)
**Figure S10** (A) 6 µm Trypan blue quenching‐ core‐shell (yellow), capsule (blue), core‐shell quenched (brown), capsule quenched (black). (B) 3 µm Trypan blue quenching‐ core‐shell (black), capsule (red), core‐ shell quenched (brown), capsule quenched (blue). (C) Rod shaped trypan blue quenching‐ core‐shell (red), capsule (black), core‐shell quenched (blue), capsule quenched (brown)
**Figure S11** Trypan blue quenching of core‐shell particles attached to J774 at 4°C: no trypan blue treatment (red), trypan blue treated (blue)
