# Supplementary material for: Tunable particles alter macrophage uptake based on combinatorial effects of physical properties
Source: Bioeng Transl Med. 2017 Jan 19;2(1):92–101. doi: 10.1002/btm2.10047 (PMC5689517; doi:10.1002/btm2.10047)
Supplement: Supplementary file 1 — Figure S1 Zetapotential of particles during LbL process. Data represented as mean ± SD (n = 3) Figure S2 TEM images of capsules. (A) 3µm sphere, (B) rod (scale bars 2 µm) Figure S3 Size distribution of 3 µm core‐shell and capsule particles measured in PBS Figure S4 Size distribution of 6 µm core‐shell and capsule particles measured in PBS Figure S5 Stability of spherical 3 µm capsules in DMEM supplemented with 10% serum. Data represented as mean ± SD (n = 3) Figure S6 Stability of rod capsules in DMEM supplemented with 10% serum. Data represented as mean ± SD (n = 3) Figure S7 Cytotoxicity of 3 µm core‐shell and capsule particles to J774 cells following 24 hr incubation. Data represented as mean ± SD (n = 3) Figure S8 Spherical 3 µm core‐shell and capsule particles after opsonization with anti‐BSA IgG antibody and incubated with secondary antibody to confirm IgG presence: core‐shell (blue), capsule (green), and negative controls incubated with BSA and secondary antibody only, core‐ shell (red) and capsule (brown) Figure S9 Rod core‐shell and capsule particles after opsonization with anti‐BSA IgG antibody and incubated with secondary antibody to confirm IgG presence: capsule (brown), core‐shell (blue), negative control incubated with BSA and secondary antibody only: core‐shell (purple) Figure S10 (A) 6 µm Trypan blue quenching‐ core‐shell (yellow), capsule (blue), core‐shell quenched (brown), capsule quenched (black). (B) 3 µm Trypan blue quenching‐ core‐shell (black), capsule (red), core‐ shell quenched (brown), capsule quenched (blue). (C) Rod shaped trypan blue quenching‐ core‐shell (red), capsule (black), core‐shell quenched (blue), capsule quenched (brown) Figure S11 Trypan blue quenching of core‐shell particles attached to J774 at 4°C: no trypan blue treatment (red), trypan blue treated (blue) [file BTM2-2-092-s001.docx]

Supplementary Figures



 **Figure S 1.** Zetapotential of particles during LbL process. Data represented as mean +/- standard deviation (n = 3).


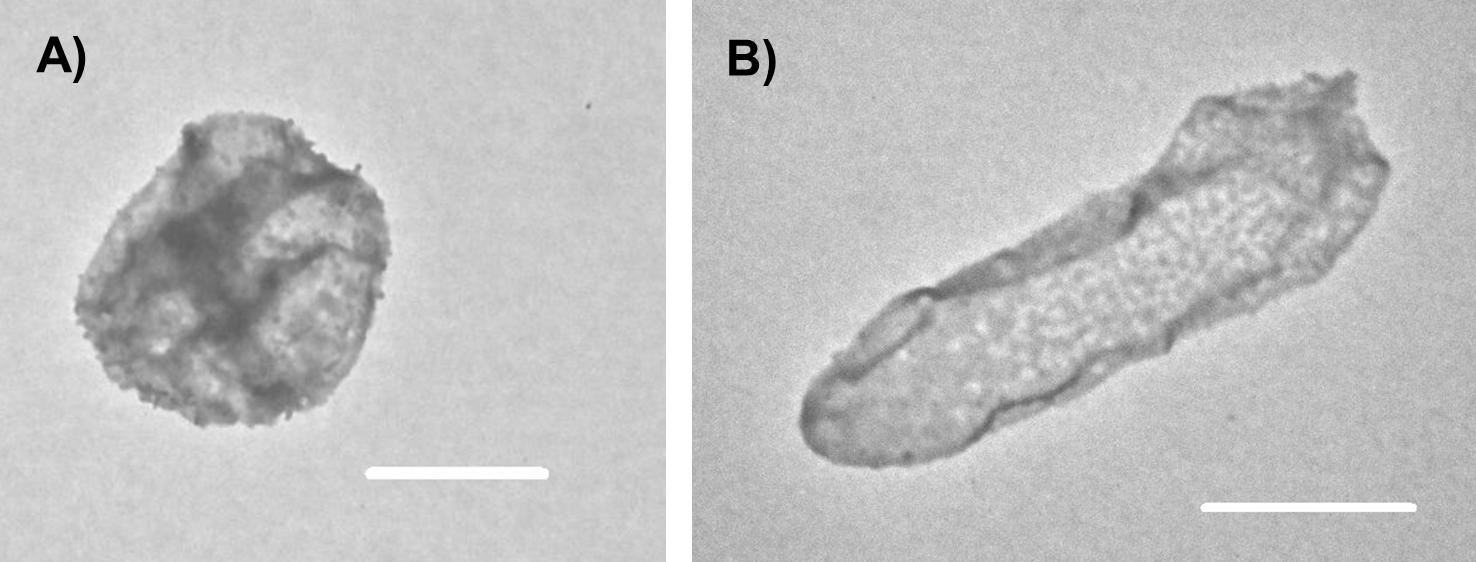


**Figure S 2.** TEM images of capsules. A) 3µm sphere, B) rod (scale bars 2 µm).


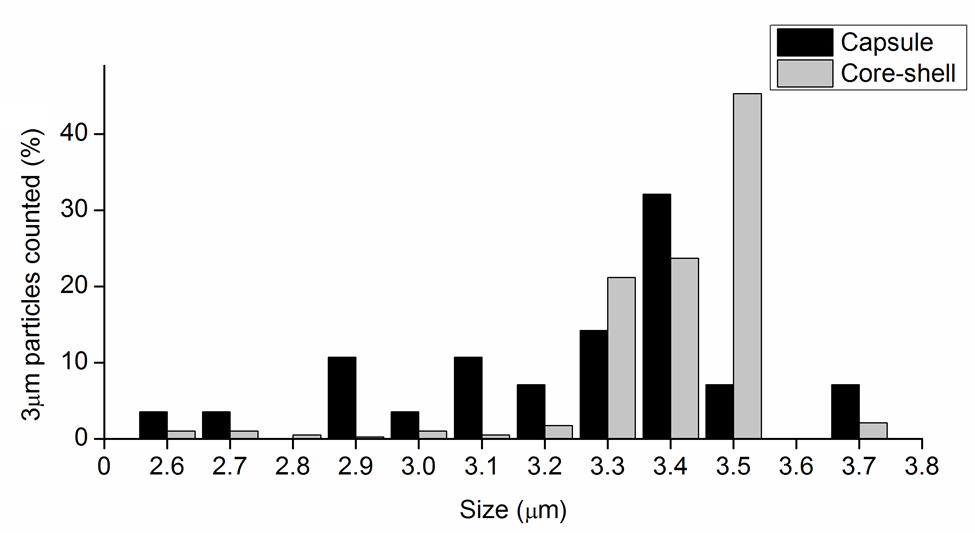


**Figure S 3.** Size distribution of 3 µm core-shell and capsule particles measured in PBS.

**
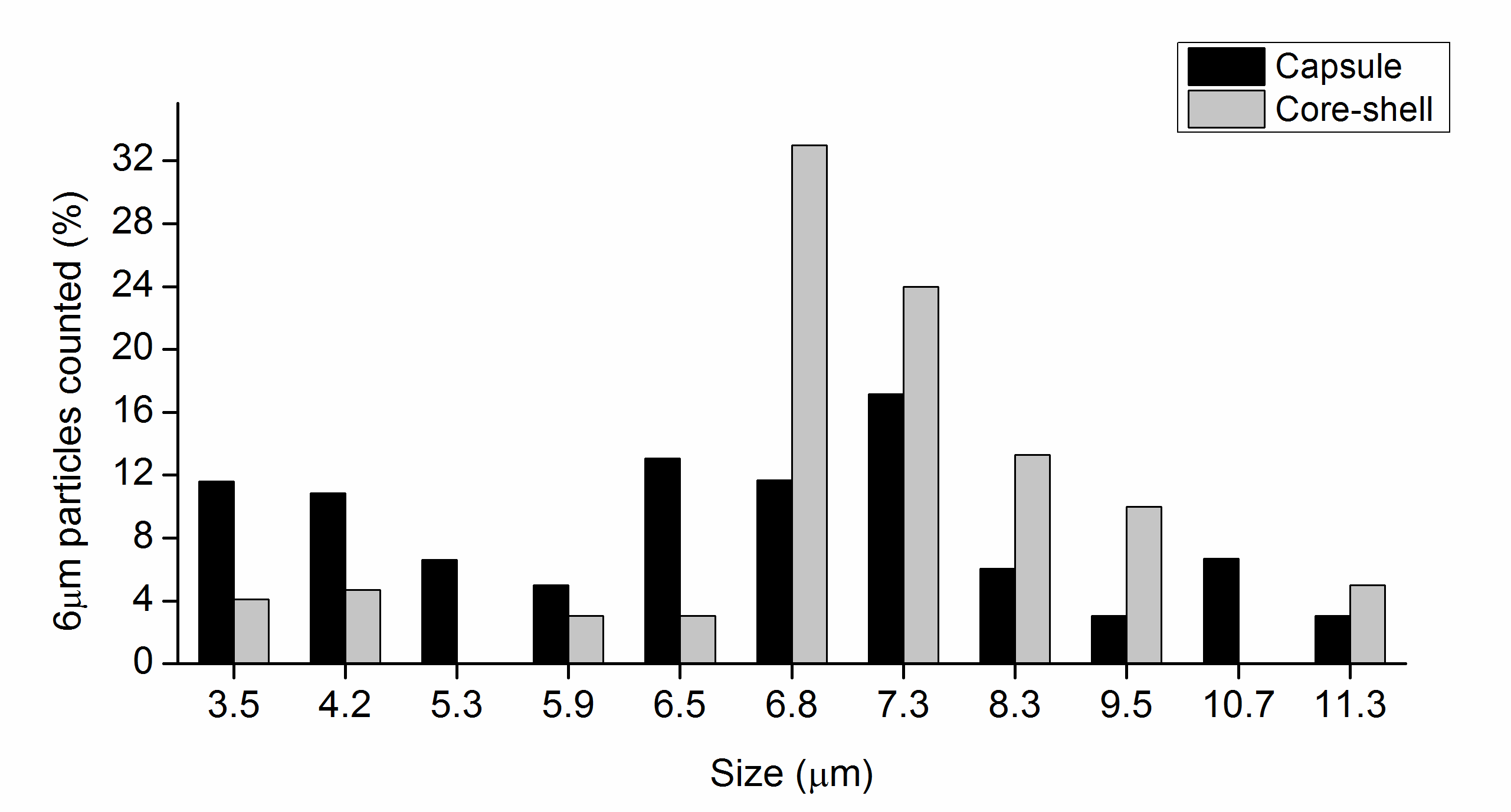
**
**Figure S 4.** Size distribution of 6 µm core-shell and capsule particles measured in PBS.


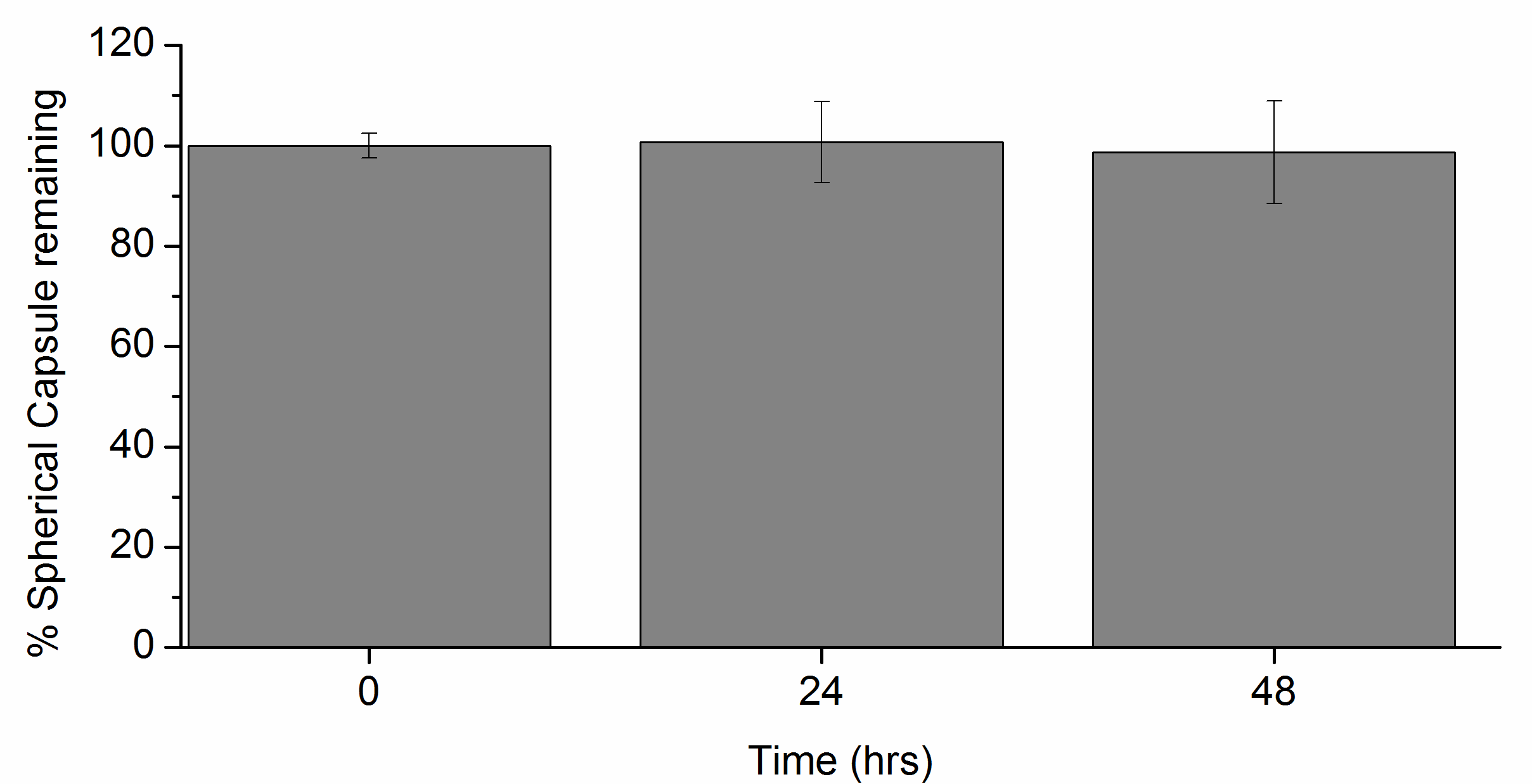


**Figure S 5.** Stability of spherical 3 µm capsules in DMEM supplemented with 10% serum. Data represented as mean +/- standard deviation (n = 3).


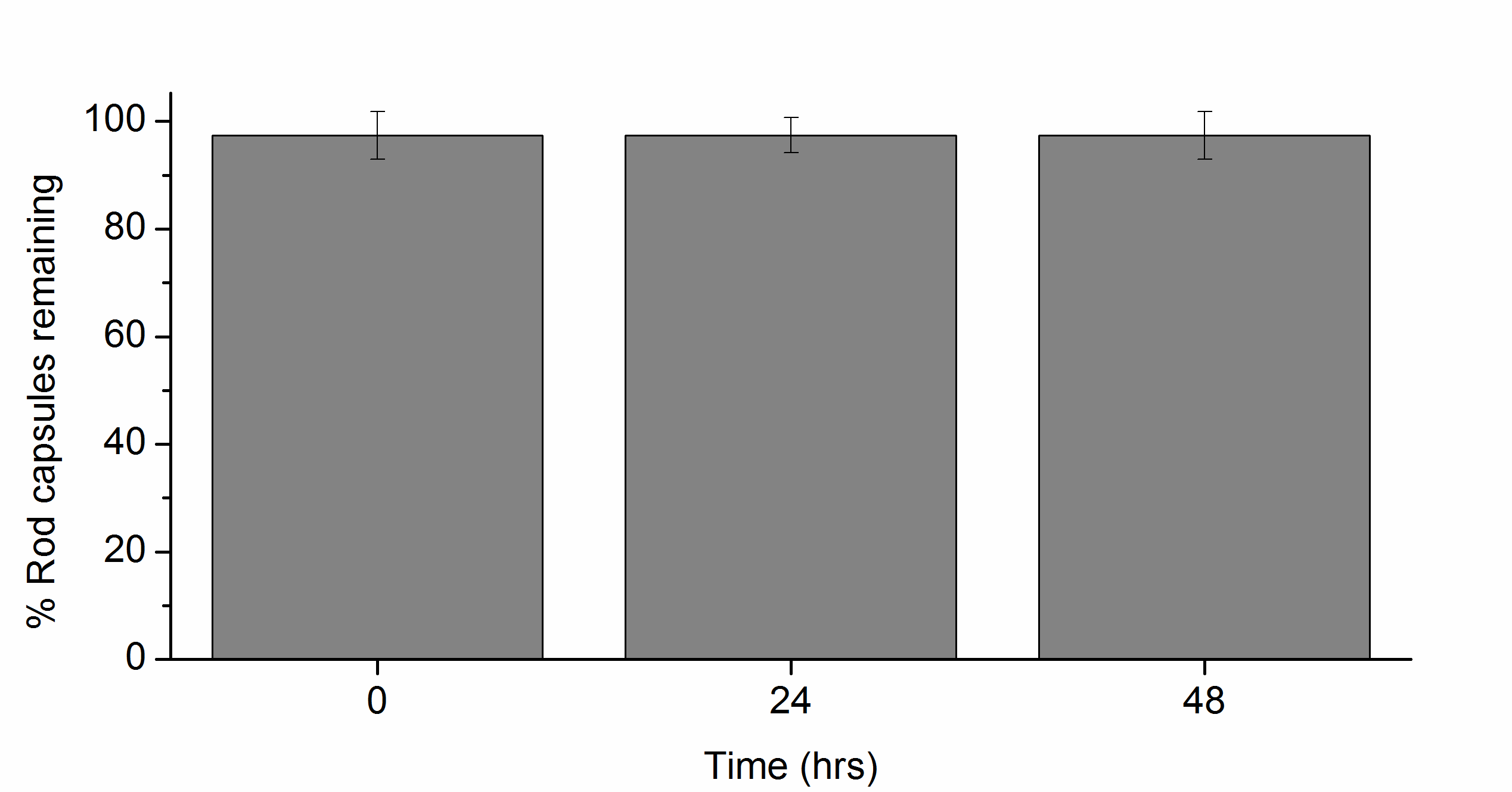
**Figure S 6.** Stability of rod capsules in DMEM supplemented with 10% serum. Data represented as mean +/- standard deviation (n = 3).

**
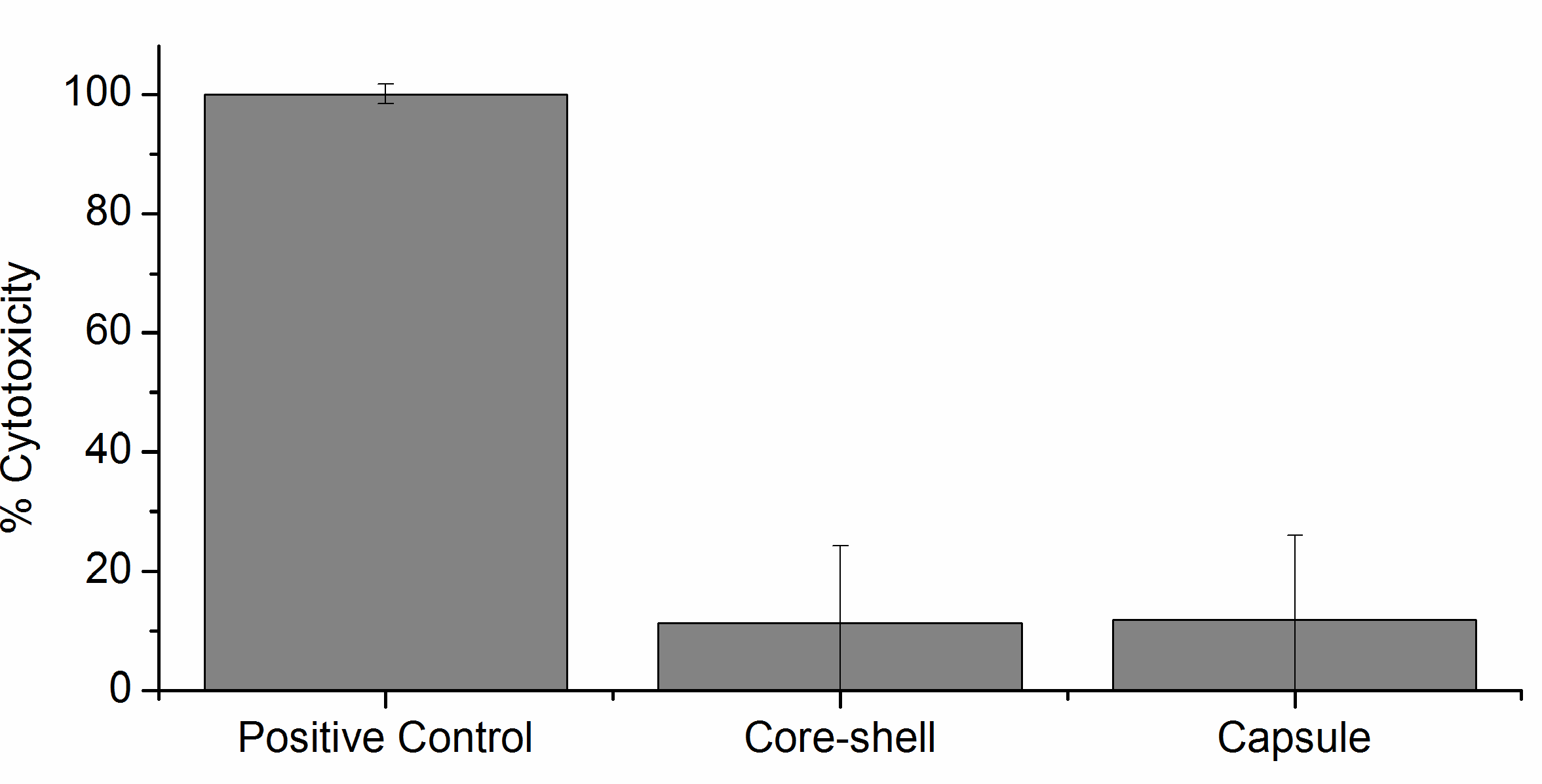
Figure S 7.** Cytotoxicity of 3 µm core-shell and capsule particles to J774 cells following 24 hrs incubation. Data represented as mean +/- standard deviation (n = 3).

**
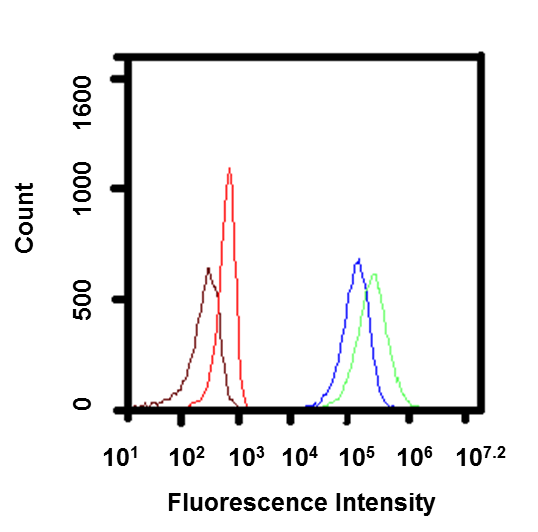
**

**Figure S 8.** Spherical 3 µm core-shell and capsule particles after opsonization with anti-BSA IgG antibody and incubated with secondary antibody to confirm IgG presence: core-shell (blue), capsule (green), and negative controls incubated with BSA and secondary antibody only, core- shell (red) and capsule (brown).


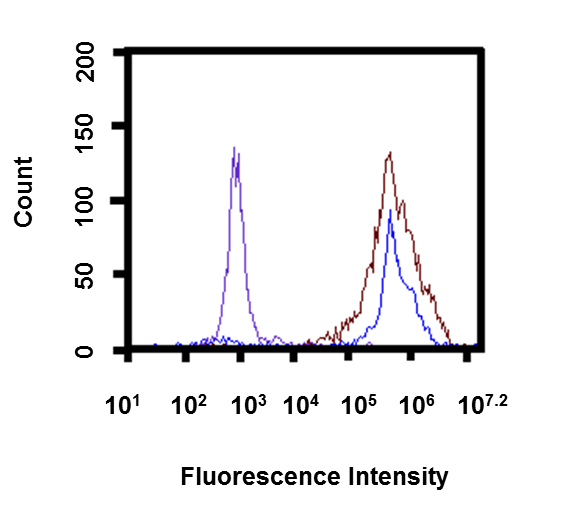


**Figure S 9.** Rod core-shell and capsule particles after opsonization with anti-BSA IgG antibody and incubated with secondary antibody to confirm IgG presence: capsule (brown), core-shell (blue), negative control incubated with BSA and secondary antibody only: core-shell (purple).


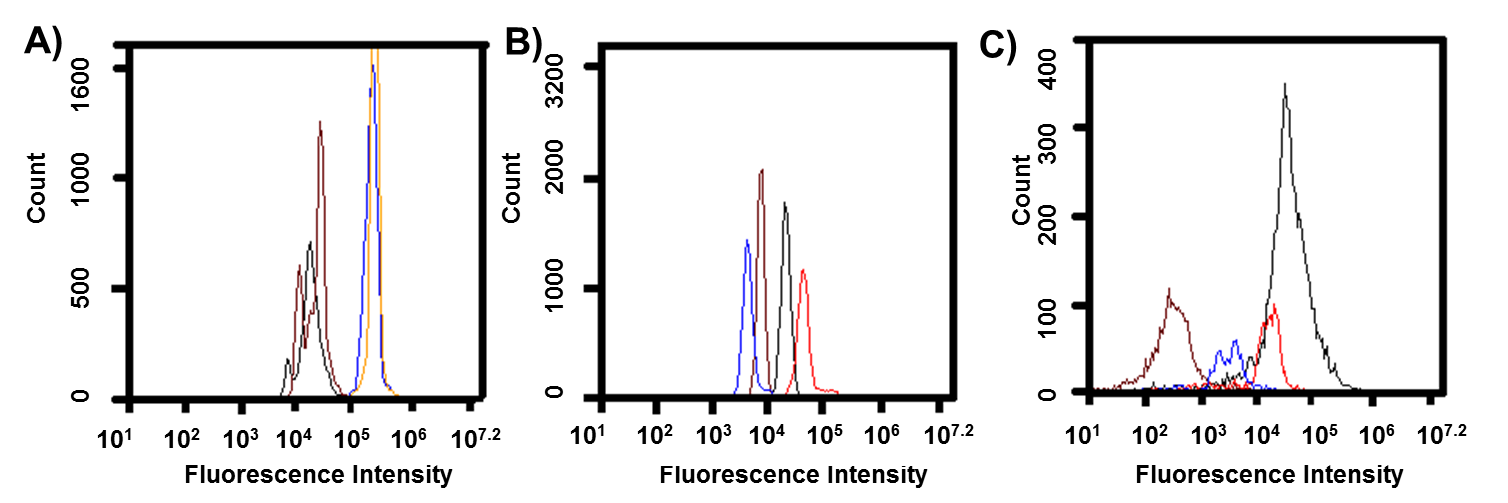


**Figure S 10.** A) 6 µm Trypan blue quenching- core-shell (yellow), capsule (blue), core-shell quenched (brown), capsule quenched (black). B) 3 µm Trypan blue quenching- core-shell (black), capsule (red), core- shell quenched (brown), capsule quenched (blue). C) Rod shaped trypan blue quenching- core-shell (red), capsule (black), core-shell quenched (blue), capsule quenched (brown).


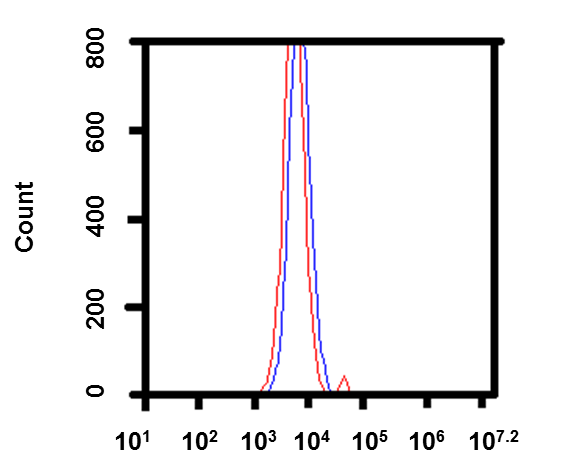


**Figure S 11.** Trypan blue quenching of core-shell particles attached to J774 at 4 ˚C: no trypan blue treatment (red), trypan blue treated (blue).

**Section1. Elasticity of hollow colloidal particles**^1^**.**

The elasticity of thin spherical shells like microcapsules has been derived analytically and is known as


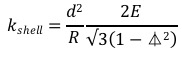


where k_shell_ is the shell spring constant, d is the thickness of the shell, R its radius, $⏃$ the poisson ratio and E the Young’s modulus of the material. Thus the stiffness of spherical capsule is

inversely proportional to the radius for the same material properties.

1. Zoldesi CI, Ivanovska IL, Quilliet C, Wuite GJL, Imhof A. Elastic properties of hollow colloidal particles. *Phys Rev E*. 2008;78(5):51401.
